# Stronger Spring Phenological Advance in Future Warming Scenarios for Temperate Species With a Lower Chilling Sensitivity

**DOI:** 10.3389/fpls.2022.830573

**Published:** 2022-05-18

**Authors:** Zhi Hu, Huanjiong Wang, Junhu Dai, Quansheng Ge, Shaozhi Lin

**Affiliations:** ^1^Key Laboratory of Land Surface Pattern and Simulation, Institute of Geographic Sciences and Natural Resources Research, Chinese Academy of Sciences, Beijing, China; ^2^University of Chinese Academy of Sciences, Beijing, China

**Keywords:** spring phenology, climate change, chilling, forcing, growth chamber experiment

## Abstract

Spring warming could induce earlier leaf-out or flowering of temperate plant species, and decreased chilling in winter has a delaying effect on spring phenology. However, the relative contribution of the decreased chilling and increased forcing on spring phenological change is unclear. Here, we analyzed the experimental data for 14 temperate woody species in Beijing, China and quantified the forcing requirements (FR) of spring phenology and chilling sensitivity (the ratio of the FR at the low chilling condition to the FR at the high chilling condition) for each species. Furthermore, using species-specific functions between the amount of chilling and FR, we established a phenological model to simulate the annual onset dates of spring events during the past 69 years (1952–2020) and in the future (2021–2099) under RCP 4.5 and RCP 8.5 climate scenarios. We also developed a novel method to quantitatively split the predicted phenological change into the effects caused by changes in forcing and those caused by changes in chilling. The results show that the FR of spring events decreased with the increase in the amount of chilling, and this relationship could be described as an exponential decay function. The basic FR (the FR at the high chilling condition) and chilling sensitivity varied greatly among species. In the 1952–2020 period, the advancing effect of increased forcing was stronger than the effect of chilling, leading to earlier spring events with a mean trend of −1.96 days/decade. In future climate scenarios, the spring phenology of temperate species would continue to advance but will be limited by the decreased chilling. The species with lower chilling sensitivities showed stronger trends than those with high chilling sensitivities. Our results suggested that the delaying effect of declining chilling could only slow down the spring phenological advance to a certain extent in the future.

## Introduction

Plant phenology refers to the periodic biological events formed by adapting to environmental conditions such as temperature, moisture, and light (Lieth, 1974). The change in plant phenology can indicate the impact of climate changes on biophysical systems. The global surface temperature has increased faster since 1970 than at any time in the previous 2,000 years (IPCC, 2021). Such a rapid climate change has altered plant phenology significantly in the past several decades. For example, 78% of leafing, flowering, and fruiting timing became earlier in 1971–2000 in Europe (Menzel et al., 2006). Similar spring phenological trends were also observed in China (Ge et al., 2015) and America (Ellwood et al., 2013). Plant phenology also affects carbon uptake (Keenan et al., 2014; Xia et al., 2015) and the water cycle (Richardson et al., 2013) of terrestrial ecosystems, and the phenological mismatch among trophic levels could contribute to community restructuring (Cleland et al., 2007; Thackeray et al., 2016). Therefore, a deeper understanding of the responses of phenology to climate change could help us to predict the future ecosystem dynamics.

In long-term phenological time series, a negative correlation between spring phenology of plants and the preseason temperature is prevalent (Wolfe et al., 2005; Wang et al., 2015, 2018), suggesting that an increase in forcing temperature can advance spring leaf unfolding or flowering date. Therefore, the earliest spring phenological models adopted the growing degree days (GDD) or thermal time to measure the impact of forcing temperature on spring phenology (Hunter and Lechowicz, 1992). In the GDD model, the developmental rate of plants is assumed to be linearly related to temperature when the temperature is above a threshold, and spring events occur when a certain GDD requirement is fulfilled. However, experimental evidence has shown that the late-autumn and winter chilling during the dormancy period also plays a key role in modulating spring phenology (Flynn and Wolkovich, 2018; Du et al., 2019; Zhang et al., 2021a), while the effect of photoperiod is only significant in a small proportion of plants (Zohner et al., 2016). Because chilling can help plants to release from endodormancy in winter (Horvath et al., 2003), plants that experienced more chilling could break bud faster under the same forcing conditions. Therefore, phenological models taking both the winter chilling and spring forcing into account can more accurately simulate the spring phenological changes (Hänninen et al., 2019). Winter warming may result in less chilling received by plants during the dormancy period and thus delay the spring phenology, but the increase in forcing temperature due to spring warming has an advancing effect. Thus, warming has dual effects on spring phenology. The relative contribution of chilling and forcing on spring phenological change is still unclear for many important species.

Since forcing and chilling are two major environmental cues modulating temperate species’ spring phenology, many studies have measured the forcing requirement (FR) and chilling requirement (CR) of various species. FR refers to the GDD required to complete the spring events under certain chilling conditions. At a fixed station, the large span of spring phenology across species implies significant interspecific differences in FR. For example, among 42 woody species at Xi’an (34°12′N, 108°57′E), China, the earliest leaf unfolding date averaged from 1963 to 2011 was March 16 (*Salix babylonica*), whereas the latest was April 21 (*Sapium sebiferum*; Dai et al., 2013). Thus, the difference in the FR of these two species reached a GDD accumulated during a 36-day period. CR is usually defined as the amount of chilling required for attaining the state of endodormancy release and could be estimated as the accumulated chilling at the date when the FR began to level off (Hänninen et al., 2019; Zhang et al., 2022). To better learn about the future phenological dynamics of different species within the community, we need to know how species’ FR and its response to chilling is related to their magnitude of phenological changes.

To investigate the interspecific difference in the FR and its sensitivity to chilling, we used experimental data on 14 temperate deciduous species from a previous study (Lin et al., 2022) and calculated species-specific functions between chilling accumulation and FR of different species. Furthermore, we developed a process-based model to simulate the past and future spring phenological changes and the relative contributions of chilling and forcing. The objectives of this study were (1) to assess the effects of climatic warming on the spring phenology of temperate species with scenario simulations, (2) to quantify the relative contribution of chilling and forcing to spring phenological changes, and (3) to identify the relationship between physiological traits (FR and its sensitivity to chilling) of species and the strength of phenological changes.

## Materials and Methods

### Twig Experiment

The experimental data were derived from a published study (Lin et al., 2022). Lin et al. (2022) carried out a twig experiment on 14 typical deciduous species (Table 1) at the Olympic Forest Park, Beijing, China (40°01′3.00″N, 116°23′2.98″E). During the two winters (1 November 2018 to 26 March 2019 and 8 November 2019 to 8 April 2020), the twigs of each species were collected every 3–7 days, resulting in twig samples with an increasing amount of chilling. In each collection, the twigs were placed in glass bottles containing tap water and then were moved to the growth chambers (day/night temperature of 25°C/15°C, and a photoperiod of 14 h light and 10 h dark). The first leaf date (FLD) or the first flowering date (FFD) of each twig in growth chambers was recorded. More detailed experimental steps could be found in Lin et al. (2022). In the present study, we investigated the FFD of three species (winter jasmine, weeping forsythia, and flowering plum) since they flowered earlier than leaf-out, while for the other 11 species, we analyzed their FLD data (Table 1).

**Table 1:** Summary of species investigated in this study.

No.	Common name	Scientific name	Life form	First spring event	Chilling model
1	Winter jasmine	*Jasminum nudiflorum*	Shrub	FFD	Eq. (1), T_1_ = 5°C
2	Rock cotoneaster	*Cotoneaster horizontalis*	Shrub	FLD	Eq. (3), T_op_ = 1°C
3	Early blooming lilac	*Syringa oblata*	Shrub	FLD	Eq. (3), T_op_ = 5°C
4	Weeping willow	*Salix babylonica*	Tree	FLD	Eq. (1), T_1_ = 5°C
5	Linden arrowwood	*Viburnum dilatatum*	Shrub	FLD	Eq. (1), T_1_ = 7°C
6	Shrub lespedeza	*Lespedeza bicolor*	Shrub	FLD	Eq. (2), T_2_ = -5°C, T_3_ = 5°C
7	Weeping forsythia	*Forsythia suspensa*	Shrub	FFD	Eq. (3), T_op_ = 5°C
8	Flowering plum	*Amygdalus triloba*	Tree	FFD	Eq. (1), T_1_ = 5°C
9	Simon poplar	*Populus simonii*	Tree	FLD	Eq. (3), T_op_ = 1°C
10	Kaido crabapple	*Malus micromalus*	Tree	FLD	Eq. (3), T_op_ = 3°C
11	Nanjing cherry	*Cerasus tomentosa*	Shrub	FLD	Eq. (3), T_op_ = 5°C
12	Maidenhair tree	*Ginkgo biloba*	Tree	FLD	Eq. (1), T_1_ = 7°C
13	Dawn redwood	*Metasequoia glyptostroboides*	Tree	FLD	Eq. (1), T_1_ = 7°C
14	Chinese ash	*Fraxinus chinensis*	Tree	FLD	Eq. (1), T_1_ = 5°C

FFD, first flowering date and FLD, first leaf date.

### Quantification of Forcing Requirement and Chilling Accumulation

The daily temperature data in Beijing (1951–2020), available from the China Meteorological Service Center,^1^ was used to calculate chilling accumulation (CA) and forcing requirements (FR). Because the hourly temperatures are not available, we generated the hourly temperatures using the sine function (Zohner et al., 2020a). For each sampling date, we first quantified the amount of CA the twigs had already experienced. The rate of chilling in response to temperature varies among species. Thus, we adopted different chilling models for each species. Three types of chilling models were used, including the threshold model (Eq. 1), range model (Eq. 2), and triangular model (Eq. 3).


(1) $$\mathrm{CU}\left(t\right)=\left\{\begin{array}{l} 1\;\;\;T_{o}\left(t\right)\leq T_{1}\\ 0\;\;\mathrm{else} \end{array}\right.\;\;$$

(2) $$\mathrm{CU}\left(t\right)=\left\{\begin{array}{l} 1\;\;\;T_{2}\leq T_{o}\left(t\right)\leq T_{3}\\ 0\;\;\;\mathrm{else} \end{array}\right.\;\;$$

(3) $$\begin{array}{l} \mathrm{CU}\left(t\right)=\left\{\begin{array}{l} \frac{T_{o}\left(t\right)-T_{\mathrm{mi}}}{T_{o}{}_{p}-T_{\mathrm{mi}}}\;T_{\mathrm{mi}}\leq T_{o}\left(t\right)\leq T_{o}{}_{p}\\ \frac{T_{\mathrm{ma}}-T_{o}\left(t\right)}{T_{\mathrm{ma}}-T_{o}{}_{p}}\;T_{o}{}_{p}\leq T_{o}\left(t\right)\leq T_{\mathrm{ma}}\\ 0\;\;\mathrm{else}\; \end{array}\right.\\ \; \end{array}$$

(4) $$\mathrm{CA}=\overset{t_{s}}{\underset{t=t_{0}}{\sum }}\mathrm{CU}\left(t\right)\;\;$$

where CA represents the amount of chilling, *t*_s_ is the last hour on the sampling date, *t*_0_ is the first hour of the day when chilling accumulation is started in the calculations (November 1). CU represents the chilling unit. T_o_(*t*) refers to the temperature at hour *t*. T_1_, T_2_, T_3_, T_op_, T_ma_, and T_mi_ are the parameters of the threshold temperature for effective chilling. For Eq. (3), the difference between T_ma_/T_mi_ and T_op_ is set to 7°C. The optimal chilling model for each species was selected based on Lin et al. (2022) and shown in Table 1.

FR was calculated as the growing degree hours (GDH) accumulated during the process of bud growth. For twigs sampled before 1 January, the accumulation of GDH started on the first day when the twigs were transferred to the growth chambers. However, for the twigs collected after 1 January, the accumulation of GDH started on 1 January, so as to include the effect of forcing temperature in natural conditions (Eq. 5).


(5) $$\begin{array}{l} \mathrm{FR}=\left\{\begin{array}{l} \overset{t_{f}}{\underset{t=t_{s}}{\sum }}\max\left(T_{g}\left(t\right)-T_{b},0\right)\;t_{s}<t_{1}\\ \overset{t_{s}}{\underset{t=t_{1}}{\sum }}\max\left(T_{o}\left(t\right)-T_{b},0\right)+\overset{t_{f}}{\underset{t=t_{s}}{\sum }}\max\left(T_{g}\left(t\right)-T_{b},0\right)\;\mathrm{else}\; \end{array}\right. \end{array}$$

where FR represented the forcing requirement of spring events; *t*_1_ is the first hour of January 1; *t*_s_ refers to the first hour on the sampling date; *t*_f_ refers to the last hour on the onset date of the first spring event (FLD or FFD) averaged from three twigs; T_o_(t) refers to the hourly temperature outdoor at hour t; T_g_(t) refers to the hourly temperature in the growth chambers; and T_b_ is the threshold temperature to accumulate GDH, which is set to 5°C following previous studies (Kramer, 1994; Peaucelle et al., 2019; Wang et al., 2020a).

### Relationship Between FR and CA

The relationship between CA and FR in natural conditions was first described as an exponential curve by Cannell and Smith (1983, 1986). Later, Murray et al. (1989) conducted an experiment on 15 tree species in Britain to examine the difference in the FR-CA curve between species. Based on the theoretical and methodological framework of Murray et al. (1989), we combined the experimental data of two winters to fit the species-specific relationship between CA and FR using an exponential function:


(6) $$\mathrm{FR}\left(\mathrm{CA}\right)=a+{\mathrm{be}}^{-c*\mathrm{CA}}$$

where a, b, and c are parameters estimated using the least-squares method, that is, the parameter values that minimize the sum of squares of the errors between the simulated FR and the observed FR in the experiment. Eq. (6) with the fitted parameters was used to simulate the past and future spring phenological changes.

To examine whether there is a significant difference in the FR and its response to CA between species, we constructed a mixed linear model with FR as the dependent variable and species, CA, and their interaction as the independent variables (fixed effects) and year as a random effect (Eq. 7):


(7) $$\ln\left(\mathrm{FR}\right)=a_{1}\mathrm{CA}+a_{2}\mathrm{Species}+a_{3}\left(\mathrm{CA}\times \mathrm{Species}\right)+a_{0}+u_{\mathrm{year}}$$

where CA refer to the CA for each species at each observation date. CA × species represents the interaction between CA and species, and a_1_, a_2_, a_3_, and a_0_ are parameters. u_year_ is the coefficient for each year. The logarithm of FR is used as a dependent variable because CA is exponentially related to FR.

### Chilling Sensitivity and Forcing Requirement

**Figure 1:**
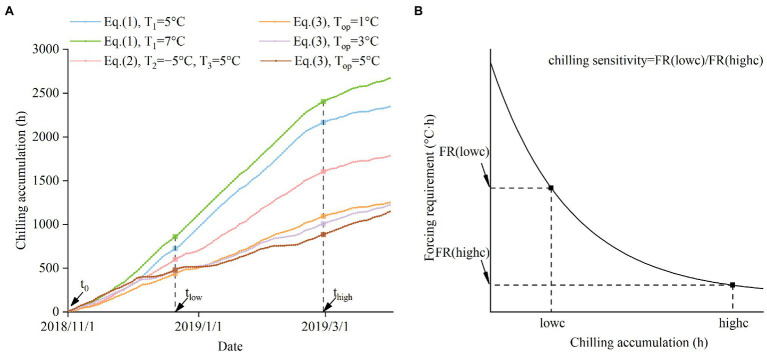
Schematic diagram of the method to calculate the chilling sensitivity (CS). **(A)** The definition of low chilling condition (lowc) and high chilling condition (highc). lowc is defined as the CA from 1 November 2018 (t_0_) to 21 December 2018 (t_low_). highc is defined as the CA from 1 November 2018 (t_0_) to 28 February 2019 (t_high_). For each species, different chilling models are used to calculate lowc and highc. **(B)** The definition of CS. CS is the ratio of the FR in lowc to the FR in highc.


**Figure 2:**
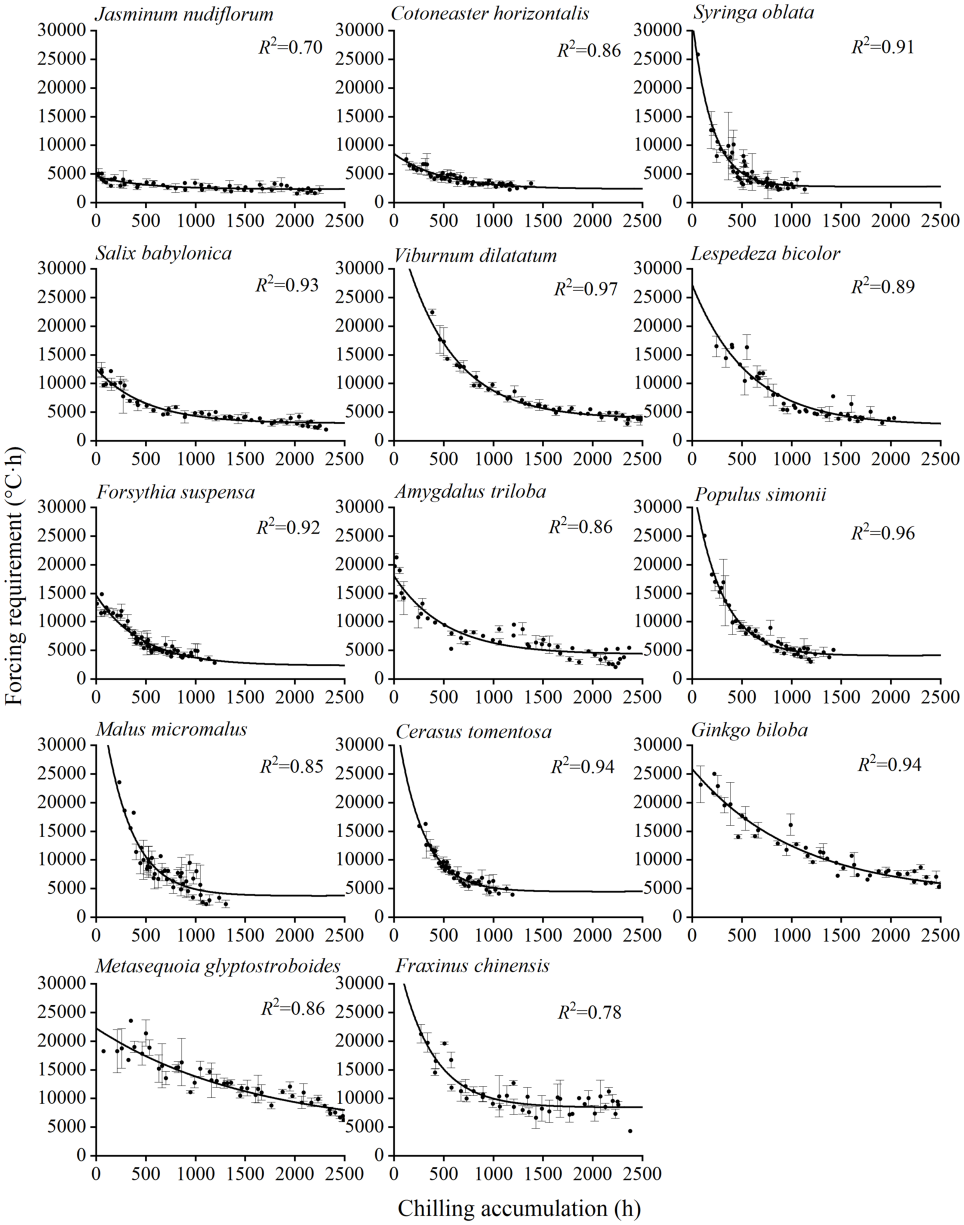
Relationship between the forcing requirement (FR) of spring events and chilling accumulation (CA) for 14 woody species. Points: observed CA and FR in the two-year experiment. Error bar: SD. An exponential function is fitted for each species, and the coefficient of determination (*R*^2^) is shown. The CA for each species is based on different chilling models (Table 1).

The rate of decrease in FR causing by the increase in chilling varies among species. To measure the interspecific difference in the effect of chilling, we defined chilling sensitivity (CS) as the ratio of the plant’s FR under low chilling conditions to the FR under high chilling conditions (Figure 1B; Eq. 8). The higher CS (a larger ratio) indicates that the plant relies more on chilling to reduce the FR. Conversely, lower CS (ratio close to 1) indicates that chilling plays a small role in regulating bud-burst date.

(8) $$\mathrm{CS}=\frac{\mathrm{FR}\left(\mathrm{lowc}\right)}{\mathrm{FR}\left(\mathrm{highc}\right)}\;$$

where FR(lowc) represents FR under low chilling conditions (lowc), and FR(highc) represents the FR under high chilling conditions (highc). FR(lowc) and FR(highc) were calculated based on Eq. (6). Using a uniform CA value of lowc and highc for all species is inappropriate because the CA values derived from different chilling models are not comparable. For example, a CA value of 720 represents a low chilling condition for *Viburnum dilatatum*, but is a relatively high chilling condition for *Forsythia suspensa* (see Figure 2). Thus, we calculated the CA values during the same period with species-specific chilling models to obtain a comparable lowc and highc (Eqs. (9), (10); Figure 1A).


(9) $$\mathrm{lowc}=\overset{t_{\mathrm{low}}\;}{\underset{t=t_{0}}{\sum }}\mathrm{CU}\left(t\right)\;\;$$

(10) $$\mathrm{highc}=\overset{t_{\mathrm{high}}\;}{\underset{t=t_{0}}{\sum }}\mathrm{CU}\left(t\right)\;\;$$

where lowc is the CA value of the low chilling condition for each species. highc is the CA value of the high chilling condition for each species. *t*_0_ is the first hour of 1 November 2018. The parameter *t*_low_ is the last hour of 21 December 2018 (corresponding to CA of 720 h calculated by Eq. 1 with T_1_ = 5°C). The parameter t_high_ is the last hour of 28 February 2019 (corresponding to CA of 2,160 h calculated by Eq. 1 with T_1_ = 5°C).

When comparing the FR among different species, we should use the FR in a constant chilling condition because the FR varies with the amount of chilling. Therefore, we used FR(highc) to represent the basic FR of each species.

### Phenological Model

We developed the phenological model based on a species-specific function between CA and FR of spring events (Eq. 6). Subsequently, we simulated the spring events of these species in the study area for the past 69 years (1952–2020) and the future 79 years (2021–2099). The future climatic data we adopted were the daily resolution and bias-corrected data simulated by the global climatic model HadGEM2-ES, covering 2011–2099 under two Representative Concentration Pathways (RCP 4.5 and 8.5), with a resolution of 0.5 × 0.5°. This dataset was available from the Fast Track input-data catalog of the Inter-Sectoral Impact Model Intercomparison Project (ISIMIP).^2^ To remove the systematic deviations of the simulated historical data from real observations, we corrected the future temperature data by adding the difference in mean temperature between the past temperature data and future temperature data during the period in common (2011–2020). Based on the past and future climate data, we found that the seasonal temperature in Beijing increased significantly at a rate of 0.34–0.52°C/decade from 1952 to 2020, and the warming is predicted to continue under both RCP 4.5 and RCP 8.5 (Supplementary Figure 1).

For each species in each year, the onset date of the spring event was simulated as the date when the GDH attained the FR estimated from the CA-FR function (Eq. 6). The regression coefficients of the phenological events against year were used to estimate the long-term trends. Furthermore, to investigate the relationship between phenological trends and CS or basic FR, we calculated the Pearson’s *r* between phenological trends and CS or basic FR of different species in the past and future periods.

Subsequently, we developed a novel method to divide the simulated phenological timing of each year into three parts (Eq. 11), including the phenological timing during the reference period (P_ref_), the change in the phenological timing caused by chilling (C), and the change in the phenological timing caused by forcing (F).


(11) $$P=C+F+P_{\mathrm{ref}}$$

The above quantitative splitting of the predicted phenological change into the effects caused by changes in forcing and those caused by changes in chilling was realized by the following steps:

1. The FR-CA curve was established for each species on the basis of the experimental results (Eq. 6).
2. Using the curve and the available temperature records, the spring phenological timing P*_i_* was calculated for each year *i* during the reference period 1961–1990.
3. The average phenological timing during the reference period was calculated by using Eq. (12).

(12) $$P_{\mathrm{ref}}=\frac{\overset{1990}{\underset{i=1961}{\sum }}P_{i}}{30}$$

4. In each year *i*, the value of FR from the FR-CA curve predicting the phenological event to occur is denoted by FR*_i_*. The mean of the FR*_i_* values during the reference period was denoted by FR_ref_ (Eq. 13). It was the mean of the forcing requirements of the phenological event during the reference period.

(13) $${\mathrm{FR}}_{\mathrm{ref}}=\frac{\overset{1990}{\underset{i=1961}{\sum }}{\mathrm{FR}}_{i}}{30}$$

5. The effect of chilling (C) was calculated as the difference between the predicted phenological timing P*_i_* and the prediction obtained for that year by assuming the constant value of FR_ref_ (Eq. 14), because the year-to-year variation in FR*_i_* is caused by the year-to-year variation of chilling.

(14) $$C=P-P\left({\mathrm{FR}}_{\mathrm{ref}}\right)$$

6. The forcing effect (F) was estimated as the difference in the phenological timing under a fixed FR (excluding the effect of CA on FR) minus the average phenological timing during the reference period (Eq. 15).

(15) $$F=P\left({\mathrm{FR}}_{\mathrm{ref}}\right)-P_{\mathrm{ref}}$$

Following Eqs. (14), (15), Eq. (11) is a mathematical necessity. Using the above methods, we calculated the interannual chilling effect and forcing effects from 1952 to 2099 and quantified the relative contribution of chilling and forcing on phenological change.

## Results

### Effect of Chilling on Forcing Requirement

The results of the mixed linear model showed that the effects of species (*p* < 0.001) and chilling (*p* < 0.001) on FR were significant, and the conditional *R*^2^ of the model reached 0.893 (Supplementary Table 1). Thus, the FR differed significantly among species and chilling conditions. The FR of spring events decreased with the increase in the CA (Figure 2). The exponential decay function fits well for the CA-FR relationship of all experimental species, with *R*^2^ ranging from 0.70 (*J. nudiflorum*) to 0.97 (*V. dilatatum*; Supplementary Table 2). Thus, the phenological model based on the exponential function between CA and FR could be used to simulate spring phenological changes.

### Chilling Sensitivity and Forcing Requirement Among Different Species

**Figure 3:**
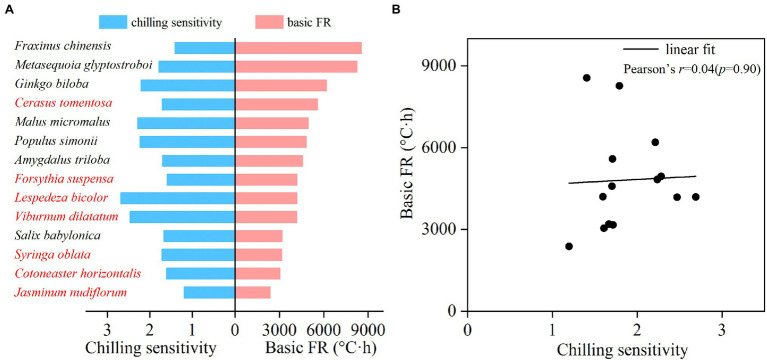
Chilling sensitivity (CS) and forcing requirement under high chilling condition (basic FR) for 14 woody species **(A)** and the relationship between CS and basic FR **(B)**. Red and black fonts represent shrubs and trees, respectively.

The basic FR and CS of each species were estimated based on the fitted CA-FR function. The CS varied greatly among 14 species, ranging from 1.19 (*J. nudiflorum*) to 2.69 (*L. bicolor*; Figure 3A). The mean CS for shrubs and trees was 1.85 and 1.90, respectively. There was no significant difference in CS between trees and shrubs (*t*-test, *p* = 0.855). The basic FR also varied significantly among species, ranging from 2383.16°C∙hours (*J. nudiflorum*) to 8556.94°C∙hours (*F. chinensis*; Figure 3A). The mean basic FR of shrubs (3824.42°C∙hours) was smaller than the trees (5799.35°C∙hours), and this difference was statistically significant (*t*-test, *p* = 0.039). Furthermore, there was no significant correlation between CS and FR across species (Figure 3B, *p* = 0.90). For example, *F. chinensis* and *J. nudiflorum* showed similar CS (1.40 and 1.19), but a 4-fold difference in the FR (8556.94 vs. 2383.16°C·hours).

### Past Phenological Change

**Figure 4:**
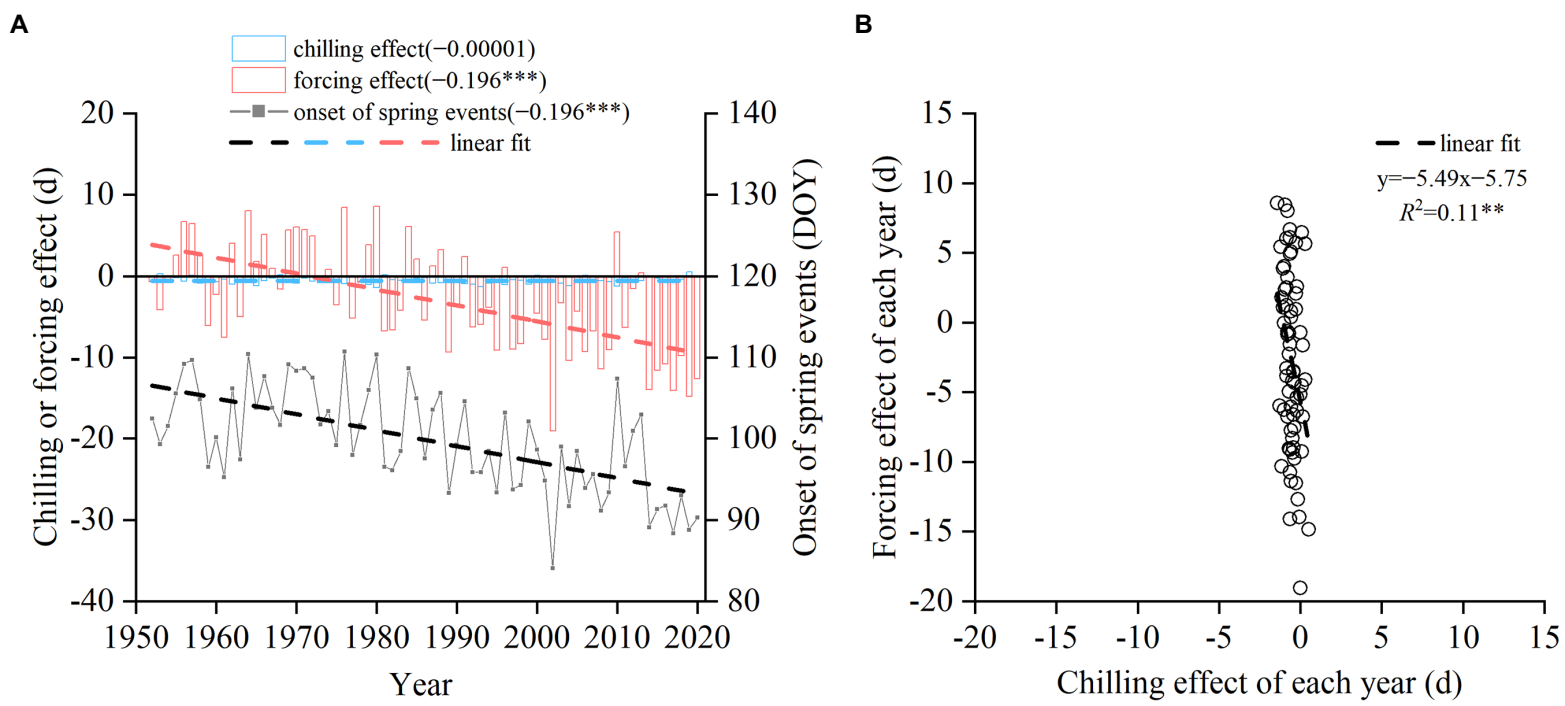
Simulated change in the timing of spring events averaged from 14 woody species from 1952 to 2020. **(A)** Annual chilling effect, forcing effect, and the onset date of spring events averaged from 14 species. DOY: day of the year. Linear trends are shown in parentheses (days/year). **(B)** Relationship between chilling effect and forcing effect across years. ***p < 0.001; **p < 0.01.

Averaged from all the species, the advancing effect of forcing increased significantly between 1952 and 2020, with a trend of −1.96 days/decade (*p* < 0.001, Figure 4A). However, the mean trend of the chilling effect was almost negligible (−0.00001 days/decade). At the interannual scale, the chilling effect was negatively correlated with the forcing effect (Figure 4B). A 1-day delay in spring phenology due to the decreased chilling corresponded to 5.49 days advance induced by the increased forcing. Thus, the delaying effect of chilling was always lower than the advancing effect of forcing.

**Figure 5:**
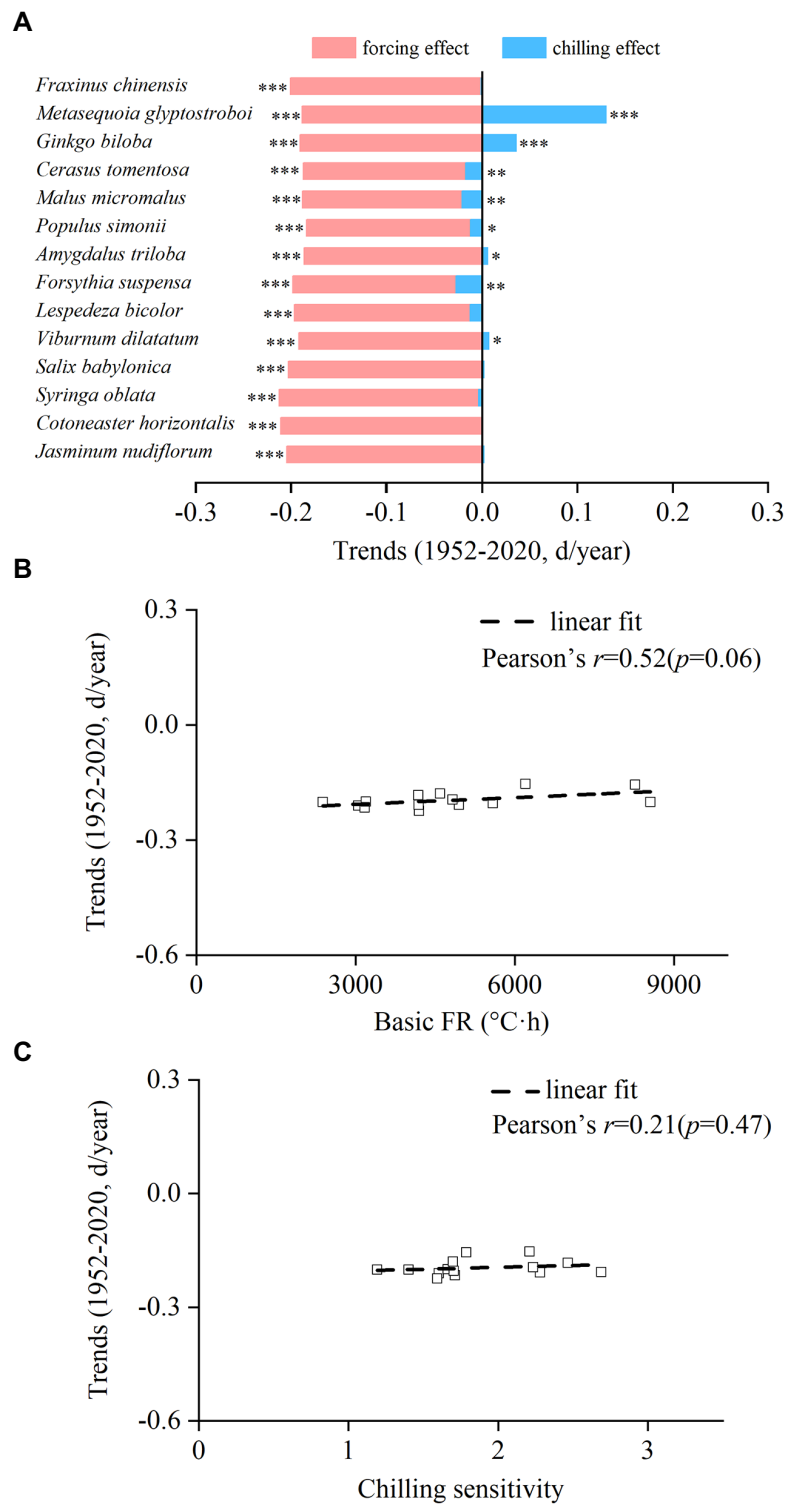
Interspecific difference in trends of the chilling effect and forcing effect on spring events from 1952 to 2020. **(A)** Trends of the chilling effect and forcing effect for each species. **(B)** Relationship between the trends of spring events and basic forcing requirement (basic FR). **(C)** Relationship between the trends of spring events and chilling sensitivity. ***p < 0.001; **p < 0.01; *p < 0.05.

The interspecific differences in the contribution of chilling and forcing to spring phenological change were obvious. The trend of the forcing effect ranged from −2.12 days/decade (*S. oblata*) to −1.83 days/decade (*P. simonii*), while the trend of the chilling effect ranged from −0.27 days/decade (*F. suspensa*) to 1.30 days/decade (*M. glyptostroboides* (Figure 5A). Because the trend of chilling effect was always weaker than the trend of forcing effect, the spring event became earlier for all species, with the trend ranging from −2.25 days/decade (*F. suspensa*) to −1.54 days/decade (*G. biloba*). The spring phenological trends during 1952–2020 were positively correlated with the CS and basic FR of each species, but the relationships were not statistically significant (Figures 5B,C).

### Future Phenological Change

**Figure 6:**
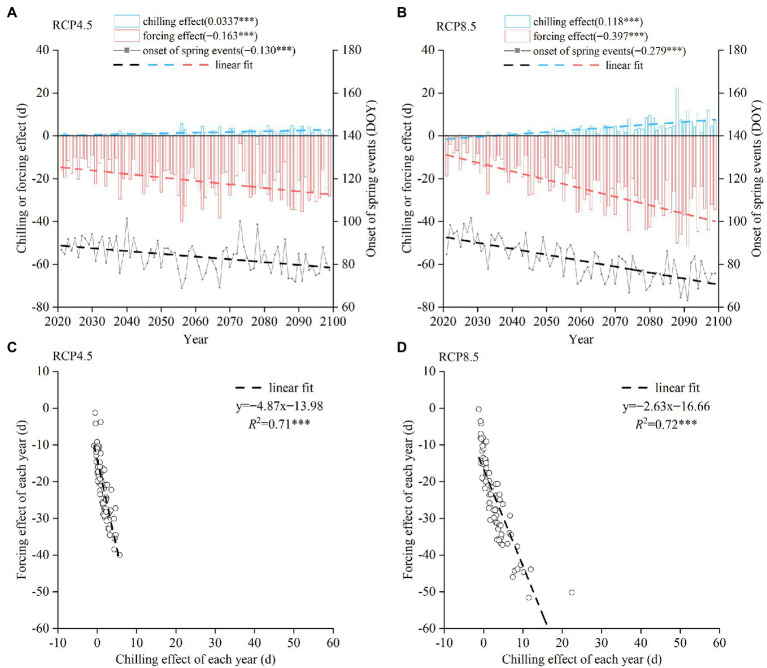
Simulated change in the spring phenophases of 14 woody species during 2021–2099. **(A,B)** Annual chilling effect, forcing effect, and the onset date of spring events averaged from 14 species under RCP 4.5 **(A)** and RCP 8.5 **(B)**. DOY: day of the year. **(C,D)** Relationship between chilling effect and forcing effect across years under RCP 4.5 **(C)** and RCP 8.5 **(D)**. ***p < 0.001.

In the future (2021–2099), the spring events of the 14 species showed an earlier trend both under RCP 4.5 or RCP 8.5. The mean trend of spring phenology under RCP 4.5 was −1.30 days/decade (Figure 6A), which was weaker than the trend under RCP 8.5 (−2.79 days/decade; Figure 6B). The delaying effect of chilling and the advancing effect of forcing were significantly stronger in the future than in the past (1952–2020). The chilling and forcing effects were still significantly negatively correlated at the interannual scale. A 1-day delay in spring phenology due to the decreased chilling corresponded to 4.87 days (RCP 4.5, Figure 6C) or 2.63 days (RCP 8.5, Figure 6D) advance caused by the increased forcing. Thus, the delaying effect of chilling reduction on spring phenology will become stronger in the future compared to past decades.

**Figure 7:**
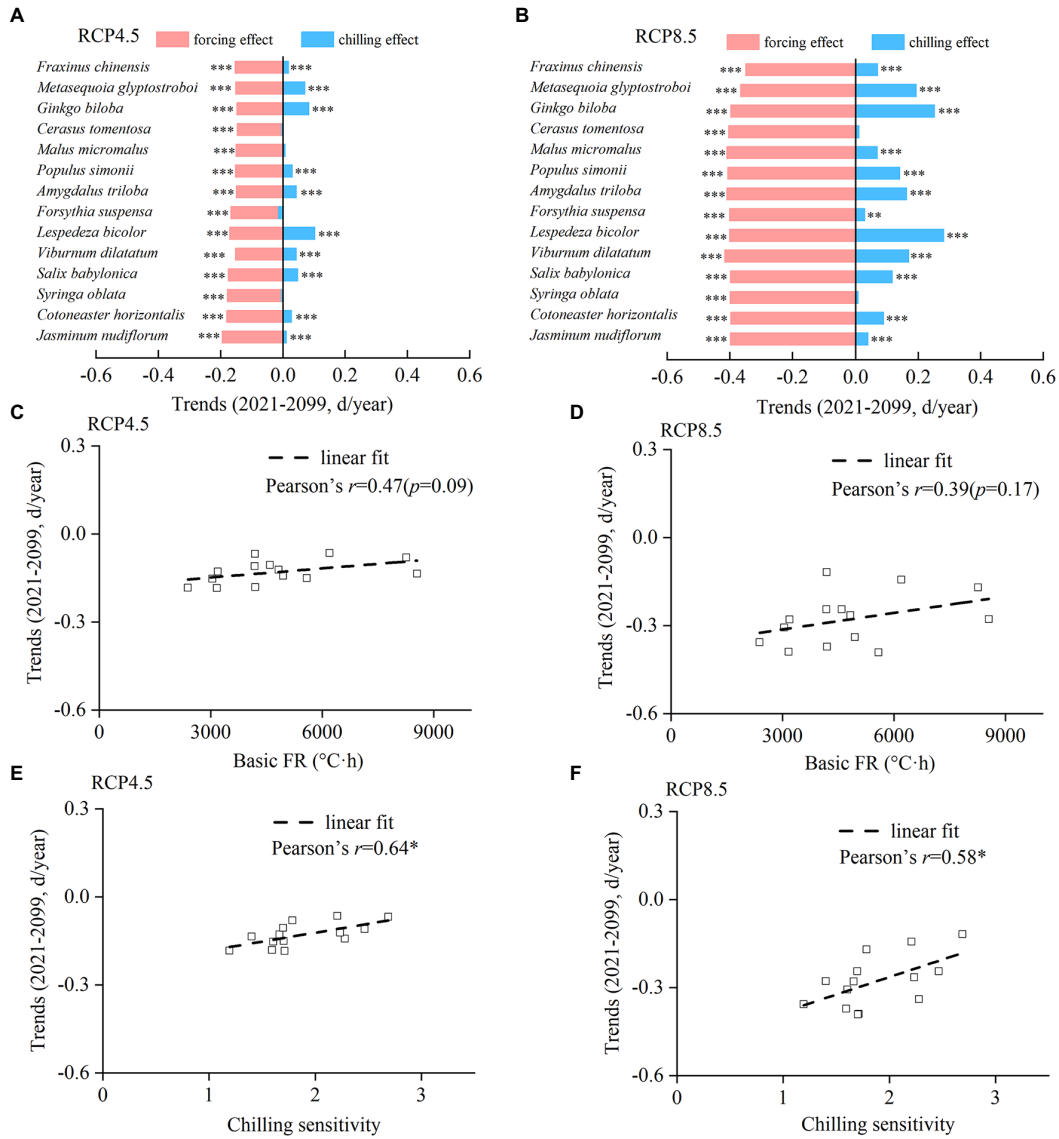
Interspecific difference in trends of the chilling effect, forcing effect, and onset date of spring events from 2021 to 2099. **(A,B)** Trends of the chilling effect and forcing effect for each species under RCP 4.5 **(A)** and RCP 8.5 **(B)**. **(C,D)** The relationship between the trends of spring events and basic forcing requirement (basic FR). **(E,F)** Relationship between the trends of spring events and chilling sensitivity. ***p < 0.001; **p < 0.01; *p < 0.05.

The trends of chilling and forcing effects in the future differed among species, resulting in different spring phenological trends (Figures 7A,B). Under RCP 4.5, spring events would become earlier in all species with a trend ranging from −1.22 days/decade (*F. chinensis*) to −0.17 days/decade (*M. glyptostroboides*). Under RCP 8.5, all species showed a significantly earlier trend ranging between −3.92 days/decade (*C. tomentosa*) and −1.18 days/decade (*L. bicolor*). Both the chilling and forcing effects were significantly stronger under RCP 8.5 compared to those under RCP 4.5 (Figures 7A,B).

Although the Pearson’s *r* between spring phenological trend and basic FR was not significant under RCP 4.5 and RCP 8.5 (Figures 7C,D), stronger spring phenological trends were found in species with lower basic FR. However, the Pearson’s *r* between spring phenological trend and CS was significantly positive (Figures 7E,F), suggesting that the advance of spring phenology in the future was more pronounced for species with lower CS.

## Discussion

### Chilling Effects on Forcing Requirements

This study confirmed that chilling played an important role in modulating the spring phenology of plants, which is consistent with the previous experimental evidence. For example, the seedlings or twigs of five subtropical trees (*Cinnamomum chekiangense*, *Liriodendron chinense*, *M. glyptostroboides*, *Phoebe chekiangensis*, and *Torreya grandis*) with longer chilling exposure showed a faster budburst rate (Zhang et al., 2021b). Pletsers et al. (2015) also found that the budburst time of two tree species (*Betula pubescens* and *Populus tremula*) was earlier with the increase in the *CA.* The controlled experiment not only could directly reflect the effect of chilling on spring phenology, but also could help to develop biologically realistic process-based models of spring phenology in trees if the experiments were explicitly designed for describing the temperature responses of the simulated processes (e.g., Zheng et al., 2021; Zhang et al., 2022). Long-term observations also showed consistent results with the controlled experiment. For instance, Fu et al. (2015b) found that FR for leaf unfolding was negatively correlated with CA for about 90% of the time series of long-term phenological observations in Europe. Similar results were found by Wang et al. (2020b), who showed that the CA-FR relationship of 30 perennial species in Europe was negative if adopting an appropriate model to measure the amount of chilling.

Although the 14 woody species, we investigated were distributed in the same region, there were significant interspecific differences in the CS and FR. This result was consistent with previous studies. For example, Man et al. (2017) found that the FR for budburst was higher for black spruce and white spruce than that for lodgepole pine and jack pine, and the rate of chilling completion was highest for black spruce and white spruce and lowest for trembling aspen and balsam poplar. As suggested by Körner and Basler (2010), most shrubs are pioneer species, which adopt an opportunistic strategy to leaf out earlier. Thus, we expect pioneer species to show lower CS and FR. Our results supported this hypothesis when comparing the FR between life forms, as tree species had higher FR than shrub species. By contrast, for chilling, our results did not support the hypothesis because the CS of trees and shrubs did not show a significant difference.

### Contribution of Chilling and Forcing to Phenological Change

Our study quantified the contribution of chilling and forcing to phenological changes of different species and found that the delaying effect of chilling was much smaller than the advancing effect of forcing from 1952 to 2020. This finding explained why long-term spring phenological changes observed on the ground are dominated by advancement (Wang et al., 2015; Menzel et al., 2020; Rosbakh et al., 2021). Furthermore, the decreased chilling caused by winter warming may limit the earlier trends of spring phenology or reduce phenological sensitivity in response to temperature (Fu et al., 2015a). For example, FFD data on *Syringa vulgaris* from 613 sites in Europe showed that the temperature sensitivity decreased significantly by 0.92 days/°C in a warming period (2004–2013) compared to that in a colder period (1963–1972; Wang et al., 2018). The future winter warming examined in the present study under RCP 4.5 (0.44°C/decade) is stronger than the spring warming (0.31°C/decade), and under RCP 8.5, warming is similar between winter and spring (both 0.77°C/decade; Supplementary Figure 1), but the chilling effect would typically be smaller than the forcing effect in all experimental species under all scenarios. Thus, in current climate conditions and future climate scenarios, the dominant signal of climate change on spring phenology is increased forcing (Ettinger et al., 2020).

### Interspecific Variation in the Future Phenological Changes

In previous studies, species with early leaf unfolding or flowering were found to have a greater phenological sensitivity to temperature and a stronger trend of advance under warming (Menzel et al., 2006; Miller-Rushing and Primack, 2008; Dai et al., 2013). Although species with lower basic FR advanced their spring phenology more in this study (Figure 5B), this relationship was not significant, possibly because the species we studied were too few to obtain a significant correlation.

The species with lower CS exhibited stronger earlier trends in their spring phenology, especially in the future. For species with low CS, the increase in the FR because of the chilling reduction is smaller, so the advance of the spring phenology is stronger than the species with higher CS. In addition, the relationship between CA and FR is nonlinear, because the sensitivity of FR to CA decreases in high chilling conditions (Figure 2). If this result could be generalized to spatial scale, we could expect that the FR of individuals distributed at high altitudes or latitudes with long winters (i.e., high chilling conditions) is not sensitive to CA. By contrast, for individuals distributed at low elevations or latitudes, FR is sensitive to CA. Thus, the delaying effect of winter warming is possibly more pronounced at low elevations or latitudes and slowed down the spring phenological advances in these areas. This explained the more uniform spring phenology on latitudinal and altitudinal gradients found in the previous studies (Vitasse et al., 2018; Cheng et al., 2021).

Species that are more sensitive to warming may be advantageous since earlier leaf-out lengthens their growing season relative to other competitors (Cleland et al., 2012). Therefore, climate warming is more favorable for species with lower CS. However, earlier spring phenology may lead to higher frost risk at the beginning of the growing season. A trend toward an earlier last frost date may result in stable or even decreased frost risk (Dai et al., 2013; Park et al., 2021). A recent study demonstrated that the late-spring frosts increased more in Europe and East Asia than in North America, and in the future, 35% and 26% of Europe’s and Asia’s forests will be increasingly threatened by frost damage (Zohner et al., 2020b).

## Conclusion

Using published experimental data relating to the effect of chilling accumulation (CA) on the forcing requirement (FR) of 14 temperate species (Lin et al., 2022), we quantified the chilling sensitivity and basic FR of each species from the exponential function between CA and FR. The results showed that the chilling sensitivities and the basic FR varied greatly among species. Furthermore, we developed a novel method to split the spring phenological change into the effects of chilling and forcing. The delaying effect of decreased chilling was lower than, and will continue to be lower than, the advancing effect of increased chilling, implying a continued advance of spring events with climate warming despite the limitation of chilling reductions. Furthermore, the species with lower chilling sensitivities showed stronger trends of spring phenology in the future than those with higher chilling sensitivities. Thus, chilling sensitivity is a key physiological trait affecting the phenological response of temperate species to warming.

## Data Availability Statement

The datasets presented in this study can be found in online repositories. The names of the repository/repositories and accession number(s) can be found in the article/Supplementary Material.

## Author Contributions

HW designed this study. ZH and SL analyzed the data. ZH and HW wrote the manuscript. QG and JD extensively revised the writing. All authors contributed to the article and approved the submitted version.

## Funding

This work was funded by the National Key R&D Program of China (grant no. 2018YFA0606102), the National Natural Science Foundation of China (41871032), the Youth Innovation Promotion Association, CAS (grant no. 2018070), and the Program for Kezhen Excellent Talents in IGSNRR, CAS (grant no. 2018RC101).

## Conflict of Interest

The authors declare that the research was conducted in the absence of any commercial or financial relationships that could be construed as potential conflicts of interest.

## Publisher’s Note

All claims expressed in this article are solely those of the authors and do not necessarily represent those of their affiliated organizations, or those of the publisher, the editors and the reviewers. Any product that may be evaluated in this article, or claim that may be made by its manufacturer, is not guaranteed or endorsed by the publisher.

## Supplementary Material

The Supplementary Material for this article can be found online at: https://www.frontiersin.org/articles/10.3389/fpls.2022.830573/full#supplementary-material

## Footnotes

^1^ http://data.cma.cn/en

^2^ https://www.isimip.org/

## References

[ref1] Cannell M. G. R. Smith R. I. (1983). Thermal time, chill days and prediction of budburst in *Picea-sitchensis*. J. Appl. Ecol. 20, 951–963. doi: 10.2307/2403139

[ref2] Cannell M. G. R. Smith R. I. (1986). Climatic warming, spring budburst and forest damage on trees. J. Appl. Ecol. 23, 177–191. doi: 10.2307/2403090

[ref3] Cheng W. Li Z. Yan L. (2021). Uniforming spring phenology under non-uniform climate warming across latitude in China. Sci. Total Environ. 762:143177. doi: 10.1016/j.scitotenv.2020.143177, PMID: 33187697

[ref4] Cleland E. E. Allen J. M. Crimmins T. M. Dunne J. A. Pau S. Travers S. E.. (2012). Phenological tracking enables positive species responses to climate change. Ecology 93, 1765–1771. doi: 10.1890/11-1912.1, PMID: 22928404

[ref5] Cleland E. E. Chuine I. Menzel A. Mooney H. A. Schwartz M. D. (2007). Shifting plant phenology in response to global change. Trends Ecol. Evol. 22, 357–365. doi: 10.1016/j.tree.2007.04.003, PMID: 17478009

[ref6] Dai J. Wang H. Ge Q. (2013). Multiple phenological responses to climate change among 42 plant species in Xi’an, China. Int. J. Biometeorol. 57, 749–758. doi: 10.1007/s00484-012-0602-2, PMID: 23114575

[ref7] Du Y. Pan Y. Ma K. (2019). Moderate chilling requirement controls budburst for subtropical species in China. Agric. For. Meteorol. 278:107693. doi: 10.1016/j.agrformet.2019.107693

[ref8] Ellwood E. R. Temple S. A. Primack R. B. Bradley N. L. Davis C. C. (2013). Record-breaking early flowering in the eastern United States. PLoS One 8, 1–9. doi: 10.1371/journal.pone.0053788, PMID: 23342001 PMC3547064

[ref9] Ettinger A. K. Chamberlain C. J. Morales-Castilla I. Buonaiuto D. M. Flynn D. F. B. Savas T.. (2020). Winter temperatures predominate in spring phenological responses to warming. Nat. Clim. Chang. 10, 1137–1142. doi: 10.1038/s41558-020-00917-3

[ref10] Flynn D. F. B. Wolkovich E. M. (2018). Temperature and photoperiod drive spring phenology across all species in a temperate forest community. New Phytol. 219, 1353–1362. doi: 10.1111/nph.15232 29870050

[ref11] Fu Y. H. Piao S. Vitasse Y. Zhao H. De Boeck H. J. Liu Q.. (2015b). Increased heat requirement for leaf flushing in temperate woody species over 1980-2012: effects of chilling, precipitation and insolation. Glob. Chang. Biol. 21, 2687–2697. doi: 10.1111/gcb.12863, PMID: 25580596

[ref12] Fu Y. H. Zhao H. Piao S. Peaucelle M. Peng S. Zhou G.. (2015a). Declining global warming effects on the phenology of spring leaf unfolding. Nature 526, 104–107. doi: 10.1038/nature15402, PMID: 26416746

[ref13] Ge Q. Wang H. Rutishauser T. Dai J. (2015). Phenological response to climate change in China: a meta-analysis. Glob. Chang. Biol. 21, 265–274. doi: 10.1111/gcb.12648, PMID: 24895088

[ref14] Hänninen H. Kramer K. Tanino K. Zhang R. Wu J. Fu Y. H. (2019). Experiments are necessary in process-based tree phenology modelling. Trends Plant Sci. 24, 199–209. doi: 10.1016/j.tplants.2018.11.006, PMID: 30528415

[ref15] Horvath D. P. Anderson J. V. Chao W. S. Foley M. E. (2003). Knowing when to grow: signals regulating bud dormancy. Trends Plant Sci. 8, 534–540. doi: 10.1016/j.tplants.2003.09.013, PMID: 14607098

[ref16] Hunter A. F. Lechowicz M. J. (1992). Predicting the timing of budburst in temperate trees. J. Appl. Ecol. 29, 597–604. doi: 10.2307/2404467

[ref17] IPCC (2021). “Summary for Policymakers,” in Climate Change 2021: The Physical Science Basis. Contribution of Working Group I to the Sixth Assessment Report of the Intergovernmental Panel on Climate Change. eds. Valérie M.-D. Panmao Z. Anna P. Sarah L. C. Clotilde P. Sophie B.. (Cambridge: Cambridge University Press) in press.

[ref18] Keenan T. F. Gray J. Friedl M. A. Toomey M. Bohrer G. Hollinger D. Y.. (2014). Net carbon uptake has increased through warming-induced changes in temperate forest phenology. Nat. Clim. Chang. 4, 598–604. doi: 10.1038/nclimate2253

[ref19] Körner C. Basler D. (2010). Phenology under global warming. Science 327, 1461–1462. doi: 10.1126/science.1186473 20299580

[ref20] Kramer K. (1994). A modeling analysis of the effects of climatic warming on the probability of spring frost damage to tree species in the Netherlands and Germany. Plant Cell Environ. 17, 367–377. doi: 10.1111/j.1365-3040.1994.tb00305.x

[ref21] Lieth H. (1974). Phenology and Seasonality Modeling. Berlin: Springer-Verlag.

[ref22] Lin S. Wang H. Ge Q. Hu Z. (2022). Effects of chilling on heat requirement of spring phenology vary between years. Agric. For. Meteorol. 312:108718. doi: 10.1016/j.agrformet.2021.108718

[ref23] Man R. Lu P. Dang Q. L. (2017). Insufficient chilling effects vary among boreal tree species and chilling duration. Agric. For. Meteorol. Plant Sci. 8:1354. doi: 10.3389/fpls.2017.01354, PMID: 28861091 PMC5559465

[ref24] Menzel A. Sparks T. H. Estrella N. Koch E. Aasa A. Ahas R.. (2006). European phenological response to climate change matches the warming pattern. Glob. Chang. Biol. 12, 1969–1976. doi: 10.1111/j.1365-2486.2006.01193.x

[ref25] Menzel A. Yuan Y. Matiu M. Sparks T. Scheifinger H. Gehrig R.. (2020). Climate change fingerprints in recent European plant phenology. Glob. Chang. Biol. 26, 2599–2612. doi: 10.1111/gcb.15000, PMID: 31950538

[ref26] Miller-Rushing A. J. Primack R. B. (2008). Global warming and flowering times in Thoreau’s Concord: A community perspective. Ecology 89, 332–341. doi: 10.1890/07-0068.1, PMID: 18409423

[ref27] Murray M. B. Cannell M. G. R. Smith R. I. (1989). Date of budburst of fifteen tree species in Britain following climatic warming. J. Appl. Ecol. 26:693. doi: 10.2307/2404093

[ref28] Park I. W. Ramirez-Parada T. Mazer S. J. (2021). Advancing frost dates have reduced frost risk among most north American angiosperms since 1980. Glob. Chang. Biol. 27, 165–176. doi: 10.1111/gcb.15380, PMID: 33030240

[ref29] Peaucelle M. Janssens I. A. Stocker B. D. Ferrando A. D. Fu Y. H. Molowny-Horas R.. (2019). Spatial variance of spring phenology in temperate deciduous forests is constrained by background climatic conditions. Nat. Commun. 10:5388. doi: 10.1038/s41467-019-13365-1, PMID: 31772185 PMC6879605

[ref30] Pletsers A. Caffarra A. Kelleher C. T. Donnelly A. (2015). Chilling temperature and photoperiod influence the timing of bud burst in juvenile *Betula pubescens* Ehrh. and *Populus tremula* L. trees. Ann. For. Sci. 72, 941–953. doi: 10.1007/s13595-015-0491-8

[ref31] Richardson A. D. Keenan T. F. Migliavacca M. Ryu Y. Sonnentag O. Toomey M. (2013). Climate change, phenology, and phenological control of vegetation feedbacks to the climate system. Agric. For. Meteorol. 169, 156–173. doi: 10.1016/j.agrformet.2012.09.012

[ref32] Rosbakh S. Hartig F. Sandanov D. V. Bukharova E. V. Miller T. K. Primack R. B. (2021). Siberian plants shift their phenology in response to climate change. Glob. Chang. Biol. 27, 4435–4448. doi: 10.1111/gcb.15744, PMID: 34101938 PMC13420798

[ref33] Thackeray S. J. Henrys P. A. Hemming D. Bell J. R. Botham M. S. Burthe S.. (2016). Phenological sensitivity to climate across taxa and trophic levels. Nature 535, 241–245. doi: 10.1038/nature18608, PMID: 27362222

[ref34] Vitasse Y. Signarbieux C. Fu Y. H. (2018). Global warming leads to more uniform spring phenology across elevations. Proc. Natl. Acad. Sci. U. S. A. 115, 1004–1008. doi: 10.1073/pnas.1717342115, PMID: 29279381 PMC5798366

[ref35] Wang H. Dai J. Rutishauser T. Gonsamo A. Wu C. Ge Q. (2018). Trends and variability in temperature sensitivity of lilac flowering phenology. J. Geophys. Res.-Biogeo. 123, 807–817. doi: 10.1002/2017JG004181

[ref36] Wang H. Dai H. Zheng J. Ge Q. (2015). Temperature sensitivity of plant phenology in temperate and subtropical regions of China from 1850 to 2009. Int. J. Climatol. 35, 913–922. doi: 10.1002/joc.4026

[ref37] Wang H. Wang H. Ge Q. Dai J. (2020a). The interactive effects of chilling, photoperiod, and forcing temperature on flowering phenology of temperate woody plants. Front. Plant Sci. 11:443. doi: 10.3389/fpls.2020.00443, PMID: 32373144 PMC7176907

[ref38] Wang H. Wu C. Ciais P. Peñuelas J. Dai J. Fu Y.. (2020b). Overestimation of the effect of climatic warming on spring phenology due to misrepresentation of chilling. Nat. Commun. 11:4945. doi: 10.1038/s41467-020-18743-8, PMID: 33009378 PMC7532433

[ref39] Wolfe D. W. Schwartz M. D. Lakso A. N. Otsuki Y. Pool R. M. Shaulis N. J. (2005). Climate change and shifts in spring phenology of three horticultural woody perennials in northeastern USA. Int. J. Biometeorol. 49, 303–309. doi: 10.1007/s00484-004-0248-9, PMID: 15592880

[ref40] Xia J. Niu S. Ciais P. Janssens I. A. Chen J. Ammann C.. (2015). Joint control of terrestrial gross primary productivity by plant phenology and physiology. Proc. Natl. Acad. Sci. U. S. A. 112, 2788–2793. doi: 10.1073/pnas.1413090112, PMID: 25730847 PMC4352779

[ref41] Zhang R. Lin J. Wang F. Delpierre N. Kramer K. Hänninen H.. (2022). Spring phenology in subtropical trees: developing process-based models on an experimental basis. Agric. For. Meteorol. 314:108802. doi: 10.1016/j.agrformet.2021.108802

[ref42] Zhang R. Lin J. Wang F. Shen S. Wang X. Rao Y.. (2021a). The chilling requirement of subtropical trees is fulfilled by high temperatures: A generalized hypothesis for tree endodormancy release and a method for testing it. Agric. For. Meteorol. 298-299:108296. doi: 10.1016/j.agrformet.2020.108296

[ref43] Zhang R. Wang F. Zheng J. Lin J. Hänninen H. Wu J. (2021b). Chilling accumulation and photoperiod regulate rest break and bud burst in five subtropical tree species. For. Ecol. Manag. 485:118813. doi: 10.1016/j.foreco.2020.118813

[ref44] Zheng J. Hänninen H. Lin J. Shen S. Zhang R. (2021). Extending the cultivation area of pecan (carya illinoinensis) toward the south in southeastern subtropical China may cause increased cold damage. Front. Plant Sci. 12:768963. doi: 10.3389/fpls.2021.768963, PMID: 34917105 PMC8669331

[ref45] Zohner C. M. Benito B. M. Svenning J. C. Renner S. S. (2016). Day length unlikely to constrain climate-driven shifts in leaf-out times of northern woody plants. Nat. Clim. Chang. 6, 1120–1123. doi: 10.1038/nclimate3138

[ref46] Zohner C. M. Mo L. Renner S. S. Svenning J. C. Vitasse Y. Benito B. M.. (2020b). Late-spring frost risk between 1959 and 2017 decreased in North America but increased in Europe and Asia. Proc. Natl. Acad. Sci. U. S. A. 117, 12192–12200. doi: 10.1073/pnas.1920816117, PMID: 32393624 PMC7275740

[ref47] Zohner C. M. Mo L. Sebald V. Renner S. S. (2020a). Leaf-out in northern ecotypes of wide-ranging trees requires less spring warming, enhancing the risk of spring frost damage at cold range limits. Glob. Ecol. Biogeogr. 29, 1065–1072. doi: 10.1111/geb.13088

