# Supplementary material for: Stronger Spring Phenological Advance in Future Warming Scenarios for Temperate Species With a Lower Chilling Sensitivity
Source: Front Plant Sci. 2022 May 18;13:830573. doi: 10.3389/fpls.2022.830573 (PMC9158521; doi:10.3389/fpls.2022.830573)
Supplement: Supplementary file 2 [file Data_Sheet_2.docx]

**SUPPLEMENTARY MATERIALS**

**Supplementary Table 1.** Results of the mixed linear model with forcing requirement (FR) as the dependent variable and species, chilling accumulation (CA), and their interaction as the independent variables.

| **Source** | **Type III Sum of Squares** | **Den DF** | **Mean Square** | **F value** | **Significance** |
| --- | --- | --- | --- | --- | --- |
| Species | 8.1205 | 641.13 | 0.6247 | 94.350 | 0.000 |
| CA | 15.3101 | 641.94 | 15.3101 | 2312.487 | 0.000 |
| Species**×**CA | 3.8695 | 641.15 | 0.2977 | 44.959 | 0.000 |

*Conditional R^2^*=0.893

**Supplementary Table 2.** Parameters of the exponential decay functions between forcing requirement of spring events and chilling accumulation for each species.

| **NO** | **Species** | ***a*** | ***b*** | ***c*** | ***R^2^*** |
| --- | --- | --- | --- | --- | --- |
| 1 | *Jasminum nudiflorum* | 2360.458 | 2234.085 | 0.002 | 0.70 |
| 2 | *Cotoneaster horizontalis* | 2389.925 | 6133.513 | 0.002 | 0.86 |
| 3 | *Syringa oblata* | 2818.695 | 29012.459 | 0.005 | 0.91 |
| 4 | *Salix babylonica* | 3068.119 | 9473.139 | 0.002 | 0.93 |
| 5 | *Viburnum dilatatum* | 3884.864 | 35631.488 | 0.002 | 0.97 |
| 6 | *Lespedeza bicolor* | 2724.300 | 24458.418 | 0.002 | 0.89 |
| 7 | *Forsythia suspensa* | 2415.243 | 12246.168 | 0.002 | 0.92 |
| 8 | *Amygdalus triloba* | 4377.486 | 13638.594 | 0.002 | 0.86 |
| 9 | *Populus simonii* | 4154.967 | 30776.894 | 0.004 | 0.96 |
| 10 | *Malus micromalus* | 3800.120 | 40000.000 | 0.004 | 0.85 |
| 11 | *Cerasus tomentosa* | 4538.056 | 32427.448 | 0.004 | 0.94 |
| 12 | *Ginkgo biloba* | 3908.842 | 22021.721 | 0.001 | 0.94 |
| 13 | *Metasequoia glyptostroboi* | 4334.263 | 17972.379 | 0.001 | 0.86 |
| 14 | *Fraxinus chinensis* | 8505.643 | 28882.141 | 0.003 | 0.78 |

a, b, c are parameters in Eq. (6). *R^2^*: coefficient of determination.





**Supplementary Figure 1.** Interannual change in the seasonal mean temperature in Beijing observed at the meteorological station (1952-2020) and simulated by the global climatic model HadGEM2-ES under RCP 4.5 and 8.5 (2021-2099). **A**, spring (March-May); **B**, summer (June-August); **C**, autumn (September-November); **D**, winter (December-February). The dashed lines show the linear fit in the past 69 years (1952-2020) and the future 79 years (2021-2099). The values in the parentheses were the linear trends of seasonal temperature (unit, °C/year). ***: *P*<0.001.
